# FNC: An Advanced Anticancer Therapeutic or Just an Underdog?

**DOI:** 10.3389/fonc.2022.820647

**Published:** 2022-02-10

**Authors:** Daria Fayzullina, Rajesh Kumar Kharwar, Arbind Acharya, Anton Buzdin, Nicolas Borisov, Peter Timashev, Ilya Ulasov, Byron Kapomba

**Affiliations:** ^1^World-Class Research Center “Digital Biodesign and Personalized Healthcare”, Sechenov First Moscow State Medical University, Moscow, Russia; ^2^Endocrine Research Lab, Department of Zoology, Kutir Post Graduate College, Chakkey, Jaunpur, India; ^3^Tumor Immunology Lab, Department of Zoology, Institute of Science, Banaras Hindu University, Varanasi, India; ^4^Department of Medical and Biological Physics, Moscow Institute of Physics and Technology, Dolgoprudny, Russia; ^5^Department of General Surgery, Parirenyatwa Group of Hospitals, Harare, Zimbabwe

**Keywords:** FNC, oncology, nucleoside (acid) analogues, azvudine, cancer

## Abstract

Azvudine (FNC) is a novel cytidine analogue that has both antiviral and anticancer activities. This minireview focuses on its underlying molecular mechanisms of suppressing viral life cycle and cancer cell growth and discusses applications of this nucleoside drug for advanced therapy of tumors and malignant blood diseases. FNC inhibits positive-stand RNA viruses, like HCV, EV, SARS-COV-2, HBV, and retroviruses, including HIV, by suppressing their RNA-dependent polymerase enzymes. It may also inhibit such enzyme (reverse transcriptase) in the human retrotransposons, including human endogenous retroviruses (HERVs). As the activation of retrotransposons can be the major factor of ongoing cancer genome instability and consequently higher aggressiveness of tumors, FNC has a potential to increase the efficacy of multiple anticancer therapies. Furthermore, FNC also showed other aspects of anticancer activity by inhibiting adhesion, migration, invasion, and proliferation of malignant cells. It was also reported to be involved in cell cycle arrest and apoptosis, thereby inhibiting the progression of cancer through different pathways. To the date, the grounds of FNC effects on cancer cells are not fully understood and hence additional studies are needed for better understanding molecular mechanisms of its anticancer activities to support its medical use in oncology.

## Introduction

Despite the best efforts of mankind, cancer remains one of the major causes of death worldwide (approximately 10 million deaths in 2020). Moreover, the proportion of cancer-associated deaths demonstrates a growing trend (https://www.who.int/ru/news-room/fact-sheets/detail/cancer, https://www.who.int/ru/news-room/fact-sheets/detail/the-top-10-causes-of-death). Many effective cancer drugs have been developed over the past decades, although none of them can guarantee to the patient long-lasting survival and protection against relapse (1). Different drugs have different molecular mechanisms, different clinical indications, and different response rates. In addition, cancers can frequently develop drug resistance, thus creating a barrier to effective tumor control (2). This stresses the importance of finding novel cancer treatment approaches, their proper combination, and their personalization (3).

**Figure 1:**
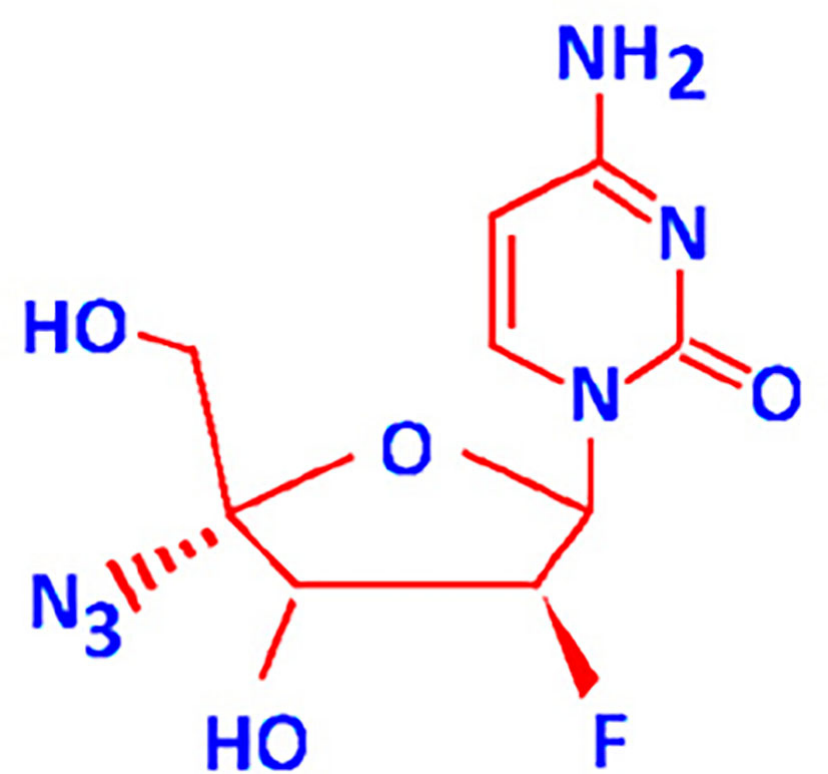
2′-Deoxy-2′-β-fluoro-4′-azidocytidine (FNC) structure.

FNC (2’-deoxy-2’-β-fluoro-4’-azidocytidine) (**Figure 1**) also known as Azvudine is a recently developed cytidine analogue. It is a new experimental drug that has been shown to be active against viruses and retrotransposons with RNA-dependent polymerase enzymes, as well as against cancer cell lines and xenografts in animal models (4–6). Anticancer activities of FNC at least in part can be linked with its inhibition of retrotransposons which had formed about 30-40% of the human DNA (7, 8). The transcriptional activation of retrotransposons including HERVs has been reported to be a consequence of multiple systemic intracellular factors (9) such as the stress response, epigenetic reprogramming, and intracellular pathways triggered by the hormones, growth factors, and cytokines (10).

The expression of retrotransposons and HERVs is tightly controlled in a normal cell with most of the elements being transcriptionally repressed (11). However, in cancer cells due to an overall deregulated epigenetic and transcriptional control, there is a strong transcriptional reactivation of retrotransposons and HERVs, which also play a role of major genomic regulatory elements (12). Many cancer types were shown to be associated with the reactivation of retrotransposons and HERVs: breast cancer (13), prostate cancer (14), melanoma (15), ovarian cancer (16), hepatocelular carcinoma (17), germ cell tumors (18), renal cell carcinoma (19, 20), leukemia (21), glioblastoma (22), and osteosarcoma (23). Retrotransposons can drive tumorigenesis through different mechanisms.

**Figure 2:**
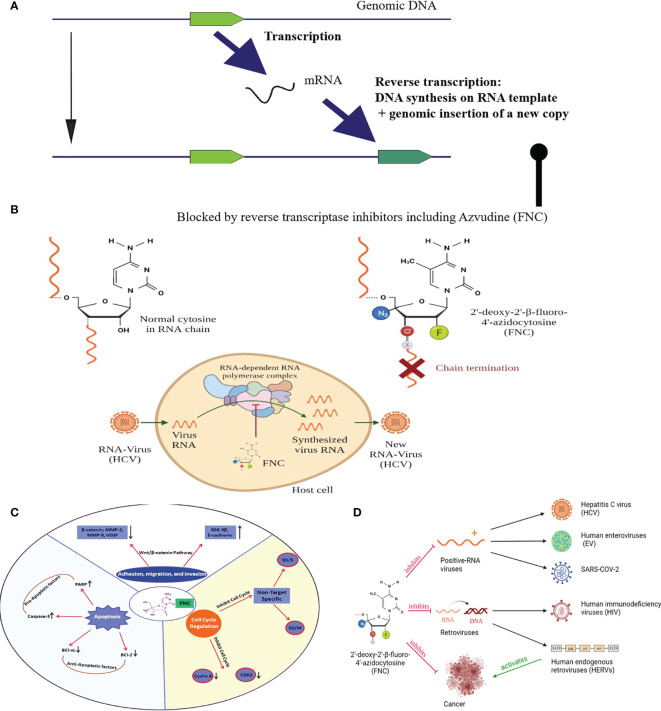
**(A)** Schematic representation of retrotransposon/HERV life cycle; **(B)** Mechanism of FNC-triggered chain termination. The 3′-OH group of FNC is unlikely to be used by polymerases for elongation of proviral RNA synthesis.; **(C)** Representation of antitumor mehanisms of FNC through multiple molecular pathways. FNC promotes apoptosis by decreasing Bcl-xL and Bcl-2 and activation of caspase-3 which further regulates proteolytic cleavage of many key proteins such as PARP. FNC inhibits the adhesion, migration and invasion of Raji and JeKo-1 cell lines by up-regulating the expression of E-cadherin and GSK-3β proteins and down-regulating the expression of proteins such as β-catenin, VEGF, MMP-2 and MMP-9. Wnt/β-catenin signaling pathway has a crucial position in the development and promotion of a wide variety of cancers. Activated Wnt/β-catenin changing the E-cadherin-β-catenin complex expression is significantly coupled with the invasiveness of tumor cells. FNC is a cell cycle-nonspecific agent which causes G1/S or G2/M phase cell cycle arrest and induces apoptosis. It inhibits cell cycle checkpoint activation (e.g., Cyclin-A binding to CDK2 permits cells to complete S phase and enter to M phase); **(D)** Graphic Abstract, depicts the possible mechanisms of FNC anticancer and antiviral activities.

Their life cycle comprises transcription of their genomic copy and further reverse transcription of the respective RNAs, *i.e.* generation of a cDNA copy on an RNA template (**Figure 2A**). The reverse-transcribed cDNA then integrates into a new genomic site to generate a new copy of retrotransposon. Most of such copies accumulated mutations and contain, if any, only interrupted/truncated non-functional open reading frames (24). However, there is a fraction of few hundred active human retrotransposons that harbor a functional reverse transcriptase (RT) gene (25) and, therefore, can mediate genomic insertion of new copies (25). Interestingly, most part of the active human retrotransposons is presented by the elements that don’t code for RT but instead utilize for their proliferation reverse transcriptase from the other elements. Indeed, the number of active non-autonomous retrotransposons of the Alu and SVA families is one or two orders higher than the number of autonomous elements with fully functional RT (26). Their functional reactivation in cancer can result in a dramatic increase of retrotransposition events, *i.e.* generation of their novel genomic copies (27). Interestingly, similar effects were observed also in other long-term stress conditions such as the retroviral infection by HIV (28).

It should be mentioned that all human retrotransposons are repetitive sequences presented in the genome by hundreds or thousands of copies (29). They totally occupy nearly 40% of the human DNA and may serve as the substrates for recombination, especially with the newly inserted copies (8). Such recombinations can occur especially frequently in cancers, where DNA repair mechanisms function abnormally (30). These recombinations lead to various genomic rearrangements and gene conversion events, including deletions, translocations, and amplifications (25).

Alternatively, transcriptional reactivation of retrotransposons in cancer can lead to expression of HERV-encoded oncogenic proteins that can influence passing through the cell cycle checkpoints, and possess fusogenic and immunosuppressive activities (31, 32). On the other hand, massive expression of multiple normally-silent retrotransposon copies may result in the appearance of novel antigens that are presented by the major histocompatibility complex (MHC) molecules. The latter can provoke specific immune response (33), which can be a specific anticancer protective mechanism.

Nowadays, there is a growing evidence that the expression of retrotransposons, especially HERVs, is connected with cancer manifestation (17). Moreover, cancer cells with stemness features and expressing HERVs may exhibit sensitivity to antiretroviral drugs treatment (10).

This review focuses on the potential action of FNC to treat aggressive tumors in combination with chemotherapeutic and/or immunotherapy regimens. We consider known and potential mechanisms of action of FNC as the anti-cancer therapeutic and their crosslinks with its antiviral activities.

## Structure of FNC

FNC is a recently developed nucleoside analogue (1). Nucleosides consist of a nucleobase and a ribose or deoxyribose sugar residue, thus showing considerable structural similarity to normal nucleotides. FNC is a 4′-C-substituted-2′-deoxynucleoside with a 3′-OH group. It mimics 2′-deoxynucleosides, and its 2′-fluoro substituent improves its stability in acidic media (34). The 4′-C-substituted 2′-deoxynucleosides retain all functional groups of 2′-deoxynucleosides. Thus, FNC can compete with the cellular internally-generated nucleotides for the incorporation into DNA and RNA strands, which results in attenuated nucleotide synthesis, and interferes with cell growth and division (35). The modification in the second position makes viral RNA-dependent polymerases more sensitive to FNC. In this case, the molecule can be incorporated into both RNA and DNA – in the second position it has fluorine, and not the functional group that determines the sugar residue type (36). FNC is also a preferred substrate for deoxycytidine kinase, and it is phosphorylated with up to 3-fold higher efficiency than its prototype, deoxycytidine (36).

## Pharmacological Mechanisms of FNC

### FNC and Positive-Strand RNA Viruses

FNC was first synthesized in 2009 among many potential therapeutic molecules for screening more specific treatments of the Hepatitis C virus (HCV) (37). It showed up to 125 times greater efficacy than the previously used treatment, and the effect was more selective (38). Since HCV often cause chronic disease that may result in liver cirrhosis or hepatocellular carcinoma, it is important to detect this infection as early as possible to start treatment. The major antiviral mechanism of FNC is thought to be the inhibition of viral replication. When FNC is incorporated into viral RNA newly synthesized by the HCV RNA-dependent RNA polymerase NS5B, it causes preliminary chain termination and, therefore, nonfunctional viral genomic RNA (**Figure 2B**).

Similarly, RNA synthesis by the RNA-dependent RNA polymerase can be interrupted for other viruses as well. For example, Na Xu et al. (6). showed for the first time that FNC can be used as an effective inhibitor for broad spectrum of human enterovirus (EV) pathogens. With high specificity and efficiency, the nucleoside analog is inserted into the positive or negative RNA strand by EV71 viral RNA-dependent RNA polymerase 3Dpol and results in truncated viral RNAs, as shown by quantitative real-time reverse transcription-PCR (RT-qPCR). The same mechanism is suggested also for the FNC antiviral activity on SARS-COV-2, another virus with the sense single-stranded RNA genome (39).

### FNC and Retroviruses

FNC also may function as the nucleoside reverse transcriptase inhibitor (NRTI), being a potentially effective agent for the treatment of retroviral infections. Being preferred targets for cellular nucleotide kinases, fluoronucleosides can serve as the good substrates for RNA and DNA polymerases. The triphosphates of nucleoside analogs compete with the cellular endogenous deoxyribonucleotides for the incorporation into DNA during replication. FNC mimics dN, and both viral and cellular replication complexes can mistakenly include it in the newly synthesized chain. The 3′-OH group of FNC is unlikely to be used by polymerases for elongation of viral DNA synthesis, and may cause immediate chain termination of replication by blocking further addition of nucleotide residues (40). Due to its chemical modifications, FNC is targeted for viral RNA-dependent polymerases, which is why it has low cytotoxicity (6).

To date, FNC is at various stages of clinical and preclinical trials for many infectious agents, and is an approved anti-HIV drug – Azvudine (6). Emerging drug-resistant viral strains as well as long-term toxicity are the main problem in current antiviral chemotherapy (41). Unlike previous NRTIs (e.g., Lamivudine), development of drug resistance against FNC requires different kind of genetic mutations. Thus, FNC could be included in an anti-AIDS treatment pipeline to overcome drug resistance issues with other drugs (42).

In addition to nanomolar activity against NRTI-resistant and multi-resistant HIV strains, it was noted that FNC is extremely potent against HIV-1 wild-type strain without obvious cytotoxicity (43). Azvudine has been also shown to be effective against HIV-2 *in vitro* (44), and demonstrates a long-lasting inhibition of HIV infection. A possible mechanism has been proposed by Sun et al. (40): in FNC-treated HIV-1 patients, FNC can restore APOBEC3 (A3G) expression in CD4+ T cells. FNC binds to the Vif-E3 ubiquitin ligase complex, enabling APOBEC3 to avoid Vif-induced ubiquitination and degradation. In turn, APOBEC3 may effectively restrict viral replication (45). In addition, FNC showed selective entry and long-term retention in HIV-1 target cells.

Nearly one-tenth of the patients with HIV are also infected with the Hepatitis B virus (HBV) due to a similar transmission path. Importantly, FNC was shown both *in vitro* and *in vivo* to restrict proliferation of human and duck hepatitis B viruses (HBV and DHBV, respectively) (46). Moreover, FNC is effective against both wild-type and lamivudine-resistant HBV clinical isolates (47). At the same time FNC showed low cytotoxicity, thus implying acceptable side effects of the treatment. For example, cytotoxicity test on the human hepatoma cell line HepG2 showed that FNC could not cause a 50% reduction of cell viability even at the concentration of 1,000 μM, which is ~200 fold higher than its physiological concentration in some previous treatments (47). Histopathological analysis also demonstrated hepatoprotective effect of FNC – less virus-induced damage to the liver was observed (5).

### FNC and Cancer

Cancer cells also can be specifically affected by FNC (**Table 1**). First, FNC, as an antiviral drug, suppresses the activity of many viruses with oncogenic effects. In addition, as a nucleotide analogue, FNC demonstrates suppression of cell growth and active proliferation of cancer cells, apparently, penetrating the synthesized nucleic acid chain and causing chain termination (49). The sensitivity to antiretroviral drugs treatment for tumor initiating (stem) cells, expressing HERVs, was demonstrated (10).Specifically, HERV-K was implemented in the maintenance and plasticity of CD133-positive melanoma stem cells. As shown by Giovavinazzo et al., treatment of lung adenocarcinoma cells A549 and hepatocarcinoma HepG2 with reverse transcriptase inhibitors such as azidothymidine (AZT) and efavirenz (EFV) decreased clonogenic, cell growth and induce apoptosis. Moreover, there are a lot of side effects on different signaling pathways of the cell, the mechanisms of which require additional research.

**Table 1:** FNC nucleoside analogues and cancer.

Sr. No.	Tumor type	Specific cell line	IC-50 Value [μM]	Reference
1.	Non-Hodgkin lymphoma	Raji	0.2	(48)
2	Non-Hodgkin lymphoma	JeKo-1	0.29	(4)
3.	B-cell non-Hodgkin lymphoma	SUDHL-6	4.55	(1)
4.	B-cell non-Hodgkin lymphoma	RL	1.74	(1)
5.	B-cell non-Hodgkin lymphoma	Granta-519	0.95	(1)
6.	Human non-small cell lung cancer	A549	1.22	(1)
7.	Acute myeloid leukemia	HL60	3.30	(1)

FNC can suppresses tumor progression by inhibiting adhesion, migration, and invasion of tumor cells in a dose-dependent manner (50). This has been shown *in vitro* and *in vivo* for non-small-cell lung cancer (NSCLC) cell line H460, and for two human aggressive non-Hodgkin lymphoma cell lines Raji and JeKo-1. Both series of experiments showed that following FNC treatment the expression levels of MMP-2, MMP-9, and VEGF were suppressed while E-cadherin expression level was increased. Later, Zhang and colleagues (51) also reported increased GSK-3β expression following FNC treatment and concluded that FNC may be considered an effective chemotherapeutic agent by regulating the invasion and metastasis of aggressive non-Hodgkin lymphomas *via* inhibition of the Wnt/β-catenin signaling pathway.

Most of the studied aspects of FNC effect on cancer cells occur in a dose- and time-dependent manner. It is thought to inhibit cell growth by suppressing expression of CDKN1A, PML, TP53INP1, TNF, SPN and LST1 proteins (4). FNC also regulates the cell cycle. It may cause cell cycle arrest of a different period at different concentrations. The gene expression assay showed that *PRNP, TP53INP1, PRKAG2, SESN2, SESN3, ERN1, CDKN1A, PML* and *TCF7L2* genes are linked with the cell cycle arrest in the G1/S or G2/M phase (4). FNC can also influence gene expression through the regulation of DNA methylation (50).

The growth and development of a tumor are regulated not only by the activity of proliferation and the rate of cell growth but also by the intensity of apoptotic processes. FNC was reported to induce apoptosis by triggering both the cell death receptor-mediated extrinsic pathway (4), and mitochondrial apoptotic pathway (50) for different types of cancers. In the first case, FNC treatment significantly increased the protein expression of Fas, FasL and TNF-α. In addition, Zhang et al. (4) found that FNC treatment also affects expression levels of some other genes that play a role in the apoptosis: *CDKN1A, PML, BIRC3, CASP10* and *TNF-α*. In the second case, treatment of H460 cells with FNC inhibited Bcl-2 expression and potentiated Cytochrome C (Cyt-C) release, Bax and caspase-3 expression.

Modulating the immune system may be another important anti-tumor mechanism of FNC and should be investigated. In a study comparing the gene-expression profiles with and without FNC treatment, significant changes in the expression of several genes associated with immunity were shown. The co-stimulatory signal during T-cell activation and genes associated with IL-2, IL-4, IL-7, IL-10 pathways has been shown based on Biocarta (4). In addition, some HERVs have proven effects on the immune system too: protein products of the translation of retroviral sequences, for example, the Gag of the HERV-K gene, affect the interaction of cancer cells with the immune system (14, 19). Retroviruses’ activity and metabolites, secreted by tumor microenvironment, inducing immunosuppressive activity along with other properties of the stem of cancer cells (10). HERVs also induces local immune checkpoint activation (19). Therefore, suppression of retroviral activity by FNC can lead to an increase in the effectiveness of immunotherapeutic strategies. Actually, targeting of the HERV-K envelope protein by CAR-T cells has already been reported as a potential immunotherapeutic approach for melanoma and other tumors (52).

Those pleiotropic effects of FNC on the cell growth, cell cycle arrest and apoptosis can be mediated by the independent reasons, or by the common functional nodes. For example, PML and CDKN1A are related to all the above processes (4). This suggests that FNC could be a potent pleiotropic molecule for treatment of multiple pathological conditions in cancer (**Figure 2C**). However, those studies may be considered fragmentary as they don’t provide comprehensive high-throughput gene expression analysis connected with the FNC effects. Therefore, a series of more in-depth studies of the FNC molecular mechanism of action on the cell cycle and apoptosis will be critical to support its potential applicability in cancer therapy.

Thus, FNC can inhibit adhesion, migration, invasion, and proliferation of tumor cells, is involved in the cell cycle arrest, immune system process and apoptosis, thereby suppressing the progression of cancer. Therefore, FNC may affect the occurrence and development of tumors through multiple molecular pathways. For most of these processes, it has been shown that the impact of FNC occurs in a dose- and time-dependent manner.

The effectiveness of FNC has been already demonstrated *in vitro* and *in vivo* for the cell lines of NSCLC and lung adenocarcinoma (50), non-Hodgkin lymphomas (49), acute myeloid leukemia (1), and for mouse xenograft models of hepatocarcinoma (H22), sarcoma (S180), and gastric carcinoma (SGC7901) (1).

At the same time, FNC has demonstrated relatively low toxicity. For example, in the experiments with mantle cell lymphoma in SCID mice (4) the low- and medium-dose FNC groups did not demonstrate significant body weight loss compared to the negative control group. Further histopathological examination of the liver and kidney tissues revealed no signs of drug toxicity.

## Clinical Trials of FNC

FNC has nanomolar activity against NRTI-resistant and multi-resistant HIV strains. This is most probably due to different mutations involved in the viral resistance against previously used NRTIs, and FNC. Indeed, Wang et al. analyzed HIV strains resistant to FNC-, or previous generation NRTI (3TC – lamivudine), and found that in 3TC-resistant viruses the dominant mutation was M184V (valine replacing methionine at position 184 in the reverse transcriptase gene), which was detected in only ~2% of FNC-resistant strains (44). FNC-resistant clones, in turn, had increased M184I mutation rate (44).

In a trial “Azvudine vs HIV-infection/AIDS, phase II (NCT04109183)”, FNC was administered in the form of a tablet medicine. This was a phase II multicenter, randomized, double-blind, double-simulation, positive control trial with 172 participants. The subjects were randomized to the treatment group of 3TC (positive control) or different doses (from 2 to 4 mg per tablet) of FNC. The background drugs (reverse transcriptase inhibitors therapy) were Efavirenz and Tenofovir disoproxil fumarate. FNC showed no serious adverse effects, exhibited desirable pharmacokinetics, and met the efficacy endpoints (39), thus forming the basis for the third phase trials.

One of such trials has started in March 2020 by HeNan Sincere Biotech in China (Phase III Clinical Study of Azvudine in Hiv-infected Treatment Naive Patients, NCT04303598). Based on the results of the previous phase, the optimal dose of 3 mg tablet of active ingredient was chosen. This is a randomized, double-blind, double-simulated, active-controlled phase III trial with 720 participants enrolled evaluating the efficacy and safety of Azvudine combined with tenofovir fumarate and efavirenz in HIV-infected treatment naive patients. FNC in combination therapy is compared with 3TC, estimated completion date is August 2022.

Alternatively, the third phase clinical trial evaluating the use of Azvudine against SARS-COV-2 started on April 2021 and was estimated to be completed in December 2021. FNC has been successful against this infection in preclinical trials (53). Later on in a randomized, open-label, controlled clinical trial of FNC in the treatment of mild and common COVID-19 (a Pilot Study) it was found that FNC treatment of mild and common COVID-19 patients may shorten the time of nucleic acid negativity conversion versus standard antiviral treatment according to the “Diagnosis and treatment program trial version 5 (or 6) guidelines” issued by the National Health Commission of China. The term was reduced by an average of 4.5 days. During phase 1 of the trial, the climbing testing showed that 6 mg of FNC was still a safe dose, and a dose of 5 mg per day was chosen for further evaluations. No drug-related adverse effects were observed in patients treated with FNC versus ~30% after treatment with standard antiviral drugs (53).

## Conclusion

Taken overall, FNC is a novel nucleoside analogue that has both antiviral and anticancer activities (**Figure 2D**). It is an effective drug for viruses like HCV, EV and SARS-COV-2 with a positive strand RNA genome. On the other hand, it belongs to the nucleoside reverse transcriptase inhibitors (NRTIs) group and may suppress HIV and most probably other reverse transcriptase containing viruses and transposable elements, including retrotransposons and HERVs. Finally, FNC shows considerable anti-cancer activity, which theoretically can be due to both cell cycle attenuation and the suppression of retrotransposons/HERVs. Whether these effects can be improved by the possible combination of FNC treatment with TK/Gancyclovir or 5FC/CD prodrug systems need to be evaluated. However, basic mechanisms which laid a foundation for FNC application are not fully understood, and additional studies are needed to elucidate the FNC activities in cancer and healthy human cells to support its medical applications other than treating viral infections.

## Author Contributions

DF, Conceptualization, Methodology, Investigation, Writing - Original Draft, Writing - Review and Editing. RK, Conceptualization, Writing - Original Draft, Visualization. AA, Conceptualization, Writing - Original Draft, Visualization. AB, Writing - Original Draft, Writing - Review and Editing. NB, Writing, Conceptualization, Visualization. PT,-Review. IU, Conceptualization, Supervision, Writing – Review. BK, Review. All authors contributed to the article and approved the submitted version.

## Funding

This work is supported by the Russian Science Foundation under grant № 21-15-00213 (IU, description of molecular mechanisms, antiviral activity of FNC, clinical trials) and grant 21-74-20066 (NB, anticancer activity of FNC).

## Conflict of Interest

Author AB is employed by OmicsWay Corp., Walnut, CA, 91789, USA.

The remaining authors declare that the research was conducted in the absence of any commercial or financial relationships that could be construed as a potential conflict of interest.

## Publisher’s Note

All claims expressed in this article are solely those of the authors and do not necessarily represent those of their affiliated organizations, or those of the publisher, the editors and the reviewers. Any product that may be evaluated in this article, or claim that may be made by its manufacturer, is not guaranteed or endorsed by the publisher.

## References

[1] Wang Q Liu X Wang Q Zhang Y Jiang J Guo X. FNC, a Novel Nucleoside Analogue Inhibits Cell Proliferation and Tumor Growth in a Variety of Human Cancer Cells. Biochem Pharmacol (2011) 81(7):848–55. doi: 10.1016/j.bcp.2011.01.001 21219886

[2] Gatenby RA Brown JS. Integrating Evolutionary Dynamics Into Cancer Therapy. Nat Rev Clin Oncol (2020) 17: (11):675–86. doi: 10.1038/s41571-020-0411-1 32699310

[3] Buzdin A Sorokin M Garazha A Glusker A Aleshin A Poddubskaya E. RNA Sequencing for Research and Diagnostics in Clinical Oncology. Semin Cancer Biol (2020) 60:311–23. doi: 10.1016/j.semcancer.2019.07.010 31412295

[4] Zhang Y Zhang R Ding X Peng B Wang N Ma F. FNC Efficiently Inhibits Mantle Cell Lymphoma Growth. PloS One (2017) 12(3):e0174112. doi: 10.1371/journal.pone.0174112 28333959 PMC5363836

[5] Yang Q Zhao X Zang L Fang X Zhao J Yang X. Anti-Hepatitis B Virus Activities of α-DDB–FNC, a Novel Nucleoside–Biphenyldicarboxylate Compound in Cells and Ducks, and its Anti-Immunological Liver Injury Effect in Mice. Antiviral Res (2012) 96: (3):333–9. doi: 10.1016/j.antiviral.2012.10.003 23098744

[6] Xu N Yang J Zheng B Zhang Y Cao Y Huan C. The Pyrimidine Analog FNC Potently Inhibits the Replication of Multiple Enteroviruses. J Virol (2020) 94(9):204–20. doi: 10.1128/JVI.00204-20 PMC7163137 32075935

[7] Schumann GG Gogvadze EV Osanai-Futahashi M Kuroki A Munk C Fujiwara H. Unique Functions of Repetitive Transcriptomes. Int Rev Cell Mol Biol (2010) 285:115–88. doi: 10.1016/B978-0-12-381047-2.00003-7 21035099

[8] Jiang Y Zong W Ju S Jing R Cui M. Promising Member of the Short Interspersed Nuclear Elements (Alu Elements): Mechanisms and Clinical Applications in Human Cancers. J Med Genet (2019) 56(10):639–45. doi: 10.1136/jmedgenet-2018-105761 30852527

[9] Buzdin AA Prassolov V Garazha AV. Friends-Enemies: Endogenous Retroviruses Are Major Transcriptional Regulators of Human DNA. Front Chem (2017) 5:35. doi: 10.3389/fchem.2017.00035 28642863 PMC5462908

[10] Giovinazzo A Balestrieri E Petrone V Argaw-Denboba A Cipriani C Miele MT. The Concomitant Expression of Human Endogenous Retroviruses and Embryonic Genes in Cancer Cells Under Microenvironmental Changes is a Potential Target for Antiretroviral Drugs. Cancer Microenviron (2019) 12(2-3):105–18. doi: 10.1007/s12307-019-00231-3 PMC6937370 31691184

[11] Suntsova M Garazha A Ivanova A Kaminsky D Zhavoronkov A Buzdin A. Molecular Functions of Human Endogenous Retroviruses in Health and Disease. Cell Mol Life Sci (2015) 72(19):3653–75. doi: 10.1007/s00018-015-1947-6 PMC11113533 26082181

[12] Nikitin D Kolosov N Murzina A Pats K Zamyatin A Tkachev V. Retroelement-Linked H3K4me1 Histone Tags Uncover Regulatory Evolution Trends of Gene Enhancers and Feature Quickly Evolving Molecular Processes in Human Physiology. Cells (2019) 8(10):1219. doi: 10.3390/cells8101219 PMC6830109 31597351

[13] Jin X Xu XE Jiang YZ Liu YR Sun W Guo YJ. The Endogenous Retrovirus-Derived Long Noncoding RNA TROJAN Promotes Triple-Negative Breast Cancer Progression *via* ZMYND8 Degradation. Sci Adv (2019) 5(3):eaat9820. doi: 10.1126/sciadv.aat9820 30854423 PMC6402854

[14] Schulz WA. Does HERV-K Represent a Potential Therapeutic Target for Prostate Cancer? Expert Opin Ther Targets (2017) 21(10):921–4. doi: 10.1080/14728222.2017.1373095 28847189

[15] Argaw-Denboba A Balestrieri E Serafino A Cipriani C Bucci I Sorrentino R. HERV-K Activation is Strictly Required to Sustain CD133+ Melanoma Cells With Stemness Features. J Exp Clin Cancer Res (2017) 36(1):1–17. doi: 10.1186/s13046-016-0485-x 28125999 PMC5270369

[16] Bannert N Hofmann H Block A Hohn O. HERVs New Role in Cancer: From Accused Perpetrators to Cheerful Protectors. Front Microbiol (2018) 9:178. doi: 10.3389/fmicb.2018.00178 29487579 PMC5816757

[17] Grabski DF Hu Y Sharma M Rasmussen SK. Close to the Bedside: A Systematic Review of Endogenous Retroviruses and Their Impact in Oncology. J Surg Res (2019) 240:145–55. doi: 10.1016/j.jss.2019.02.009 PMC9306217 30933828

[18] Chan SM Sapir T Park SS Rual JF Contreras-Galindo R Reiner O. The HERV-K Accessory Protein Np9 Controls Viability and Migration of Teratocarcinoma Cells. PloS One (2019) 14(2):e0212970. doi: 10.1371/journal.pone.0212970 30818388 PMC6394991

[19] Panda A de Cubas AA Stein M Riedlinger G Kra J Mayer T. Endogenous Retrovirus Expression is Associated With Response to Immune Checkpoint Blockade in Clear Cell Renal Cell Carcinoma. JCI Insight (2018) 3(16):e121522. doi: 10.1172/jci.insight.121522 PMC6141170 30135306

[20] Siebenthall KT Miller CP Vierstra JD Mathieu J Tretiakova M Reynolds A. Integrated Epigenomic Profiling Reveals Endogenous Retrovirus Reactivation in Renal Cell Carcinoma. EBioMedicine (2019) 41:427–42. doi: 10.1016/j.ebiom.2019.01.063 PMC6441874 30827930

[21] Deniz O Ahmed M Todd CD Rio-Machin A Dawson MA Branco MR. Endogenous Retroviruses are a Source of Enhancers With Oncogenic Potential in Acute Myeloid Leukaemia. Nat Commun (2020) 11(1):3506. doi: 10.1038/s41467-020-17206-4 32665538 PMC7360734

[22] Yuan Z Yang Y Zhang N Soto C Jiang X An Z. Human Endogenous Retroviruses in Glioblastoma Multiforme. Microorganisms (2021) 9(4):764. doi: 10.3390/microorganisms9040764 33917421 PMC8067472

[23] Ho XD Nguyen HG Trinh LH Reimann E Prans E Kõks G. Analysis of the Expression of Repetitive DNA Elements in Osteosarcoma. Front Genet (2017) 8:193. doi: 10.3389/fgene.2017.00193 29250102 PMC5714928

[24] Kazazian HH Jr. Moran JV. Mobile DNA in Health and Disease. N Engl J Med (2017) 377(4):361–70. doi: 10.1056/NEJMra1510092 PMC5980640 28745987

[25] Hancks DC Kazazian HH Jr. Active Human Retrotransposons: Variation and Disease. Curr Opin Genet Dev (2012) 22(3):191–203. doi: 10.1016/j.gde.2012.02.006 22406018 PMC3376660

[26] Mills RE Bennett EA Iskow RC Luttig CT Tsui C Pittard WS. Recently Mobilized Transposons in the Human and Chimpanzee Genomes. Am J Hum Genet (2006) 78(4):671–9. doi: 10.1086/501028 PMC1424692 16532396

[27] Scott EC Devine SE. The Role of Somatic L1 Retrotransposition in Human Cancers. Viruses (2017) 9(6):131. doi: 10.3390/v9060131 PMC5490808 28561751

[28] Jones RB Song H Xu Y Garrison KE Buzdin AA Anwar N. LINE-1 Retrotransposable Element DNA Accumulates in HIV-1-Infected Cells. J Virol (2013) 87(24):13307–20. doi: 10.1128/JVI.02257-13 PMC3838212 24089548

[29] Garazha A Ivanova A Suntsova M Malakhova G Roumiantsev S Zhavoronkov A. New Bioinformatic Tool for Quick Identification of Functionally Relevant Endogenous Retroviral Inserts in Human Genome. Cell Cycle (2015) 14(9):1476–84. doi: 10.1080/15384101.2015.1022696 PMC4612461 25853282

[30] Scott EC Gardner EJ Masood A Chuang NT Vertino PM Devine SE. A Hot L1 Retrotransposon Evades Somatic Repression and Initiates Human Colorectal Cancer. Genome Res (2016) 26(6):745–55. doi: 10.1101/gr.201814.115 PMC4889970 27197217

[31] Kassiotis G Stoye JP. Making a Virtue of Necessity: The Pleiotropic Role of Human Endogenous Retroviruses in Cancer. Philos Trans R Soc Lond B Biol Sci (2017) 372: (1732):20160277. doi: 10.1098/rstb.2016.0277 28893944 PMC5597744

[32] Lemaître C Tsang J Bireau C Heidmann T Dewannieux M. A Human Endogenous Retrovirus-Derived Gene That can Contribute to Oncogenesis by Activating the ERK Pathway and Inducing Migration and Invasion. PLoS Pathog (2017) 13: (6):. doi: 10.1371/journal.ppat.1006451 PMC5501692 28651004

[33] Smith CC Selitsky SR Chai S Armistead PM Vincent BG Serody JS. Alternative Tumour-Specific Antigens. Nat Rev Cancer (2019) 19(8):465–78. doi: 10.1038/s41568-019-0162-4 PMC6874891 31278396

[34] Yang Q Kang J Zheng L Wang XJ Wan N Wu J. Synthesis and Biological Evaluation of 4-Substituted Fluoronucleoside Analogs for the Treatment of Hepatitis B Virus Infection. J Med Chem (2015) 58(9):3693–703. doi: 10.1021/jm5012963 25905540

[35] Liu W Zhang L Zhou H Yang C Miao Z Zhao Y. Synthesis of Novel Nucleoside Analogue Phosphorothioamidate Prodrugs and In Vitro Anticancer Evaluation Against RKO Human Colon Carcinoma Cells. Nucleosides Nucleotides Nucleic Acids (2013) 32(4):161–73. doi: 10.1080/15257770.2013.770523 24001190

[36] Klumpp K Kalayanov G Ma H Le Pogam S Leveque V Jiang WR. 2'-Deoxy-4'-Azido Nucleoside Analogs are Highly Potent Inhibitors of Hepatitis C Virus Replication Despite the Lack of 2'-Alpha-Hydroxyl Groups. J Biol Chem (2008) 283(4):2167–75. doi: 10.1074/jbc.M708929200 18003608

[37] Smith DB Kalayanov G Sund C Winqvist A Pinho P Maltseva T. The Design, Synthesis, and Antiviral Activity of 4'-Azidocytidine Analogues Against Hepatitis C Virus Replication: The Discovery of 4'-Azidoarabinocytidine. J Med Chem (2009) 52(1):219–23. doi: 10.1021/jm800981y 19055482

[38] Smith DB Kalayanov G Sund C Winqvist A Maltseva T Leveque VJ-P. The Design, Synthesis, and Antiviral Activity of Monofluoro and Difluoro Analogues of 4′-Azidocytidine Against Hepatitis C Virus Replication: The Discovery of 4′-Azido-2′-Deoxy-2′-Fluorocytidine and 4′-Azido-2′-Dideoxy-2′, 2′-Difluorocytidine. J med Chem (2009) 52(9):2971–8. doi: 10.1021/jm801595c 19341305

[39] Yu B Chang JJST Therapy T. Azvudine (FNC): A Promising Clinical Candidate for COVID-19 Treatment. Signal Transduct Target Ther (2020) 5: (1):1–2. doi: 10.1038/s41392-020-00351-z 33040075 PMC7547293

[40] Sun L Peng Y Yu W Zhang Y Liang L Song C. Mechanistic Insight Into Antiretroviral Potency of 2′-Deoxy-2′-β-Fluoro-4′-Azidocytidine (FNC) With a Long-Lasting Effect on HIV-1 Prevention. J Med Chem (2020) 63(15):8554–66. doi: 10.1021/acs.jmedchem.0c00940 32678592

[41] Wang Q Li Y Song C Qian K Chen C-H Lee K-H. Synthesis and Anti-HIV Activity of 2′-Deoxy-2′-Fluoro-4′-C-Ethynyl Nucleoside Analogs. Bioorg Med Chem Lett (2010) 20: (14):4053–6. doi: 10.1016/j.bmcl.2010.05.090 PMC2915458 20542430

[42] Wang Q Li Y Song C Qian K Chen CH Lee KH. Synthesis and Anti-HIV Activity of 2'-Deoxy-2'-Fluoro-4'-C-Ethynyl Nucleoside Analogs. Bioorg Med Chem Lett (2010) 20(14):4053–6. doi: 10.1016/j.bmcl.2010.05.090 PMC2915458 20542430

[43] Li H Dou H Zhang Y Li Z Wang R Chang J. Studies of the Interaction Between FNC and Human Hemoglobin: A Spectroscopic Analysis and Molecular Docking. Spectrochim Acta A Mol Biomol Spectrosc (2015) 136:416–22. doi: 10.1016/j.saa.2014.09.051 25448944

[44] Wang R-R Yang Q-H Luo R-H Peng Y-M Dai S-X Zhang X-J. Azvudine, a Novel Nucleoside Reverse Transcriptase Inhibitor Showed Good Drug Combination Features and Better Inhibition on Drug-Resistant Strains Than Lamivudine. vitro (2014) 9(8):e105617. doi: 10.1371/journal.pone.0105617 PMC4140803 25144636

[45] Sadeghpour S Khodaee S Rahnama M Rahimi H Ebrahimi D. Human APOBEC3 Variations and Viral Infection. Viruses (2021) 13(7):1366. doi: 10.3390/v13071366 34372572 PMC8310219

[46] Zheng L Wang Q Yang X Guo X Chen L Tao L. Antiviral Activity of FNC, 2'-Deoxy-2'-Beta-Fluoro-4'-Azidocytidine, Against Human and Duck HBV Replication. Antivir Ther (2012) 17(4):679–87. doi: 10.3851/IMP2094 22452880

[47] Zhou Y Zhang Y Yang X Zhao J Zheng L Sun C. Novel Nucleoside Analogue FNC is Effective Against Both Wild-Type and Lamivudine-Resistant HBV Clinical Isolates. Antivir Ther (2012) 17(8):1593–9. doi: 10.3851/IMP2292 22910281

[48] Zhang Y Cheng X Huang G Dong J Wang C Jiang H-h. Effect of New Nucleoside Analogue FNC on Proliferation, Apoptosis and Expressions of Bcl-6, PRDM1, C-myc in Cell Line Raji. J Zhengzhou University (2013) 4, 450–4

[49] Zhang Y Wang C-P Ding X-X Wang N Ma F Jiang J-H. FNC, a Novel Nucleoside Analogue, Blocks Invasion of Aggressive non-Hodgkin Lymphoma Cell Lines *via* Inhibition of the Wnt/β-Catenin Signaling Pathway. Asian Pacific J Cancer Prev (2014) 15(16):6829–35. doi: 10.7314/APJCP.2014.15.16.6829 25169533

[50] Jing X Niu S Liang Y Chen H Wang N Peng Y. FNC Inhibits Proliferation and Metastasis of Non-Small-Cell Lung Cancer in Vivo and In Vitro. (2021). doi: 10.21203/rs.3.rs-219276/v1

[51] Zhang Y Wang CP Ding XX Wang N Ma F Jiang JH. FNC, a Novel Nucleoside Analogue, Blocks Invasion of Aggressive non-Hodgkin Lymphoma Cell Lines via Inhibition of the Wnt/beta-Catenin Signaling Pathway. Asian Pac J Cancer Prev (2014) 15(16):6829–35. doi: 10.7314/apjcp.2014.15.16.6829 25169533

[52] Balestrieri E Argaw-Denboba A Gambacurta A Cipriani C Bei R Serafino A. Human Endogenous Retrovirus K in the Crosstalk Between Cancer Cells Microenvironment and Plasticity: A New Perspective for Combination Therapy. Front Microbiol (2018) 9:1448. doi: 10.3389/fmicb.2018.01448 30013542 PMC6036167

[53] Ren Z Luo H Yu Z Song J Liang L Wang L. A Randomized, Open-Label, Controlled Clinical Trial of Azvudine Tablets in the Treatment of Mild and Common COVID-19, A Pilot Study. Adv Sci (Weinh) (2020) 7(19):2001435. doi: 10.1002/advs.202001435 PMC7404576 35403380

